# Direct anterior versus direct lateral hip approach in total hip arthroplasty with the same perioperative protocols one year post fellowship training

**DOI:** 10.1186/s13018-023-03716-6

**Published:** 2023-03-19

**Authors:** Asim M. Makhdom, William J. Hozack

**Affiliations:** 1 Department of Orthopaedic Surgery, King Abdulaziz University, 7441 Al Mortada Street, Jeddah, 22252 Saudi Arabia; 2 Adult Reconstruction, Rothman Institute, Thomas Jefferson University, 925 Chestnut Street Floor 5, Philadelphia, PA 19107 USA

## Abstract

**Background:**

Variable results have been reported regarding the clinical outcomes in Total hip arthroplasty (THA) based on the surgical approach. The aim of this study is to compare the clinical outcomes between Direct anterior (DA) and direct lateral (DL) approaches in THA when performed immediately after fellowship training.

**Methods:**

During the 1st year of practice, all consecutive patients who underwent THA via DA and DL hip approaches were retrospectively investigated. Patients’demographics, diagnosis, American society of Anesthesiology (ASA) score, route of anesthesia, length of hospital stay (LOS), leg length discrepancy (LLD), radiographic parameters, operative time, number of opioids refills postoperatively, and complications were collected and compared between the two groups. The short form of Hip Disability and Osteoarthritis Outcome score, Joint Replacement (HOOS, JR) was prospectively collected pre and postoperatively. The minimum follow-up period was 2 years.

**Results:**

Forty patients in DA group and 38 patients in DL group were included. No statistically significant difference was found between the two groups in terms of demographics, diagnosis, ASA scores, route of anesthesia at the time of THA, postoperative radiographic parameters, LOS, LLD, opioid refills and HOOS scores (*p* > 0.05). Patients in the DA group had shorter operative time (83 ± 17 min) when compared to the DL group (93 ± 24 min) (*p* = 0.03). No major complications were found except for one early deep infection patient in DL group.

**Conclusion:**

Both DA and DL approaches resulted in satisfactory outcomes in THA when performed by a fellowship trained surgeon.

## Background

Primary total hip arthroplasty (THA) is one of the most successful surgical procedures in last century [1]. Up to date, there is no consensus on which surgical approach in THA offers superior results over another. Over the past decade, the direct anterior (DA) hip approach has been widely marketed in United States for its superiority in providing rapid postoperative recovery and rehabilitation [2]. However, various studies have examined the functional outcomes and complications rates of DA hip approach and have shown variable and not consistent results [3–5]. There are several reasons that can lead to these conclusions and often overlooked in some studies [6, 7] such as surgeon’s experience, prior fellowship training and learning curve while performing DA hip approach. To the best of our knowledge, there are no studies have examined the functional outcomes and complication rates between the DA and direct lateral (DL) hip approach when performed by a fellowship trained surgeon in both approaches during the 1st year of practice. The primary goal of this study is to report patients’ outcomes and complication rates in two cohorts of patients who underwent THA via DA and DL hip approaches by the same surgeon and same perioperative protocols. Secondary goals were to compare hospital length of stay (LOS), number of opioid refills and postoperative radiographic parameters.

## Patients and methods

After obtaining institutional ethics board approval, a retrospective study design was conducted. During the 1^st^ year of practice and after fellowship with extensive training in both DA and DL hip approaches at high volume orthopedic institute, all consecutive patients who underwent THA via DA hip approach (DA group) and DL hip approach (DL group) were investigated. All these procedures were performed by the same surgeon (AM). All consecutive patients during the 1st five months of practice were performed via DL approach and then were performed via DA approach for remaining months during the 1st year of practice. This order of surgical approach selection was due to the unavailability of anterior approach equipment during the 1st 5 months of practice.

Patients’ basic demographics, diagnosis, American society of Anesthesiology (ASA) score, route of anesthesia (spinal versus general), length of hospital stay, leg length discrepancy (LLD), femoral stem alignment, acetabular component position, number of opioids refills postoperatively, complications, operative time and the need of using walking aids at 1 and 6 weeks postoperatively were collected and compared between the two groups. The short form of Hip Disability and Osteoarthritis Outcome score, Joint Replacement (HOOS, JR) [8] was prospectively collected at the following intervals; one week preoperatively, 6 weeks and at 6 months postoperatively. Patients who had a history of dementia, bilateral staged THA, hip pathology other than primary or secondary osteoarthritis, psychiatric illness, chronic opioid use, previous surgery in the involved hip and had less than 2 years of follow-up after THA were excluded from the study. A total of 108 patients were initially reviewed. Thirty of these were excluded; 11 patients had a primary diagnosis of displaced femoral neck fracture (8 of these underwent hemiarthroplasty and 3 underwent THA), 4 patients had lost to follow-up (2 in DL group and 2 in the DA group) after one year of surgery, 2 patients died after 18 months due to complications related to COVID infection, 5 patients had bilateral staged THA, 4 patients had prior hip surgeries [childhood hip procedures (n = 3) and hip arthroscopy (n = 1)], 2 patients had a history of dementia, one patient had a history of psychiatric illness (schizophrenia) and one patient had a history of chronic opioid use. This left 78 patients (DA group = 40 and DL group = 38) who were eligible to be included in the study. The DA hip approach was performed while patients on supine position and on a regular operating room table without using traction and as described previously by post ZD et al. [9]. The DL hip approach was performed while patients on a lateral decubitus position and as previously described by Petis S et al. [10]. All patients had cementless acetabular component and cementless femoral stems with proximal porous coating. All patients received 1 g (g) of paracetamol, 200 mg (mg) of celecoxib (or 15 mg meloxicam if allergic to celecoxib), 5 mg of oxycodone and 200 mg of gabapentin within one hour prior to surgery. All patients received prophylactic 2 g of intravenous (IV) cefazolin antibiotic 30 to 60 min prior to the surgical incision and additional doses given for 24 h. IV tranexamic acid of 1 g was administrated prior to the incision and another 1 g was given at the time of skin closure. In-hospital medications included standing doses of IV 30 mg of Ketoralc every 8 h, 1 g of paracetamol every 8 h, 200 mg of gabapentin twice a day and 5 mg of oxycodone every 6 h as needed until the hospital discharge. All patients received the following regimen for pain control after hospital discharge; standing doses of paracetamol (1 g every 8 h) for 2 weeks, standing doses of celecoxib 200 mg twice a day or (meloxicam 15 mg once a day if allergic to celecoxib) for 2 weeks, gabapentin 200 mg twice a day for 2 weeks and only 10 tablets of oxycodone (5 mg every 6 h as needed). Any oxycodone refill request during post-operative discharge period was recorded. The online Prescription Drug Monitoring Program (PDMP) was also checked to see if patients had any additional opioids refills elsewhere. All patients received Aspirin 81 mg twice for 4 weeks postoperatively as chemical deep venous thrombosis prophylaxis and had pneumatic compression devices applied while in hospital. The radiographic method to determine the LLD was as follows; measuring the perpendicular distance from the proximal corner of the lesser trochanter to the horizontal reference line (most distal aspect of the obturator foramens) in both hips. The difference between the ipsilateral and contralateral hip was measured and determined to be the LLD. A LLD more than 5 mm was considered as an outlier. The Widmer technique was used to determine the cup inclination angle and anteversion angles [11]. Cup inclination angles below 30 or above 50 or cup anteversion angles lower than 5 or higher than 25 were considered outliers [12]. All radiographic measurements in this study were performed using digital images after adjusting for the magnification differences. Two arthroplasty fellowship trained surgeons performed the measurements separately. Each performed the analysis twice on 2 separate occasions. In case there was a discrepancy between the measurements, observers then subsequently discussed the measurements until they reached a consensus. All patients were encouraged to ambulate during the 1^st^ few hours after surgery and followed standard physical therapy protocol without any restrictions. Patients who were discharged the same day or stayed overnight were grouped as (less than 24 h stay) and patients who stayed more than one night were grouped as (more than 24 h stay). The mean follow-up time was 30.1 months (range from 24 to 39 months).

### Statistical analysis

Descriptive statistics in form of means, range and standard deviations were utilized. A *student -T test* was used to compare two independent means. The *two tailed Fisher exact tests* was utilized to compare proportions between categorical variables. The statistical package for the social sciences (Inc., Chicago, IL, USA) version 28.0 was utilized for the statistical work.

## Results

A total of 78 hips (patients) were eligible and included in our analysis. Of these, 40 patients were in the DA group and 38 patients were in the DL group. There was no statistically significant difference between the two groups regarding age, primary diagnosis, Body Mass Index (BMI), route of Anesthesia (Spinal versus general), gender, laterality and ASA score (Table 1).

**Table 1:** Patients’ demographics in both direct anterior and direct lateral groups

Variables	Direct anterior group (N = 40)	Direct lateral group (N = 38)	P value
Mean Age ± SD (range)	64 ± 9.6	64.3 ± 11	0.8
Mean Body mass index ± SD	31.1 ± 4.4	30.5 ± 5.3	0.5
Spinal/general anesthesia	31/9	27/11	0.34
Gender (female/male)	27/13	21/17	0.19
Primary hip osteoarthritis/secondary hip osteoarthritis	39/1	34/4	0.16
ASA score	ASA 1 = 19 ASA2 = 20 ASA3 = 1	ASA1 = 12 ASA2 = 20 ASA3 = 6	0.07
Site (right/left)	24/16	19/19	0.2

DA group had a statistically significant shorter mean operative time (83 ± 17 min) when compared to DL group (93 ± 24 min) with *p* value = 0.03. There was no difference in HOOS scores preoperatively and at 6 week and 6 months postoperatively between the groups (*p* = 0.6 and *p* = 0.8, respectively). No statistically significant differences were found between the two groups with mean postoperative LLD and/or number of outliers (*p* = 0.2), mean cup anteversion angle and/or outliers (*p* = 0.7), hospital Length of stay (*p* = 0.1) and the need of walking aids at one and six weeks postoperatively (*p* = 0.2 and *p* = 0.5, respectively). Patients in the DA group had a higher mean cup inclination angle (46.4 ± 7.1 degrees) compared to the DL group (42.6 ± 5.5 degrees) with *p* = 0.013. However, there was no statistically significant difference between the two groups with regard the number of cup inclination outliers (15% in group A and 8% in group B, *p* = 0.4). There were 6 patients (15%) in the DL group who asked for opioid refills postoperatively compared to 3 patients (7%) in the DA group with P value = 0.2. There were 2 patients (5%) who appeared to have varus femoral stem alignment in the DA group compared to 1 patient (2.6%) in the DL group with *p* value = 0.1. Table 2.

**Table 2:** Clinical and radiographic outcomes of primary total hip arthroplasty in both direct anterior (DA) and direct lateral (DL) groups

Variables	DA group (N = 40)	DL group (N = 38)	P value
Mean Operative time in minutes ± SD	83 ± 17	93 ± 24	0.03
Mean Preoperative HOOS JR ± SD	37.1 ± 13.4	41.4 ± 12.5	0.15
Mean 6 weeks postoperative HOOS JR ± SD	63.9 ± 18.8	61.8 ± 15.6	0.6
Mean 6 months postoperative HOOS JR ± SD	86 ± 11.6	85 ± 15.9	0.8
Mean inclination angle ± SD	46.4 ± 7.1	42.6 ± 5.5	0.013
Number of outliers for cup inclination angle	6(15%)	3(8%)	0.48
Mean anteversion angle ± SD	18.1 ± 6.6	19.1 ± 6.3	0.4
Number of outliers for cup anteversion angle	5(12%)	6(15%)	0.7
Mean leg length discrepancy (LLD) in millimeters (mm) ± SD	3.2 ± 2.5	3.7 ± 2.8	0.3
Number of outliers in LLD (> 5 mm)	3(7%)	6(15%)	0.2
Femoral stem alignment	Neutral:38 Varus: 2	Neutral:37 Varus:1	0.1
Narcotics refill	3 patients (7%)	6 patients(15%)	0.2
Required walking aids at one week post-surgery	28 patients (70%)	30 patients (78%)	0.2
Required walking aids at 6 weeks post-surgery	3 patients(7.5%)	4 patients (10%)	0.5
Length of hospital stay (less than 24 h/ more than 24 h)	16(40%)/24(60%)	13(34%)/25(66%)	0.1

One Intraoperative complication occurred in the DA group. This patient had intraoperative femoral stem perforation with the tip of first broach. This was identified immediately, a prophylactic cable was added and subsequent broaching was resumed with no postoperative consequences had occurred or modifications in the physical therapy protocol were required postoperatively. There were other minor complications related to surgical approach in the DA group: temporary numbness at the lateral femoral cutaneous nerve (n = 8), and temporary groin pain that resolved within 6 months (n = 3). Similarly, the DL group had complications related to surgical approach: 3 patients had a persistent positive Trendelenburg gait postoperatively that was resolved within 6 months in all patients. In both groups, delayed wound healing at the proximal incision was encountered but with higher frequency in the DA group (n = 5) when compared with the lateral group (n = 1). All these healed within 4 weeks postoperatively using local wound care. Table 3.

**Table 3:** Reported complications in of primary total hip arthroplasty in both direct anterior (DA) and direct lateral (DL) groups

List of complications	Anterior hip approach (N = 40)	Lateral hip approach (N = 38)
Femoral canal perforation	One patient had intraoperative small posteromedial femoral canal perforation (with the tip of the 1^st^ broach). It was identified immediately and prophylactic cable was added. No consequences or modifications on the physical therapy protocol/weight bearing during postoperative period had occurred	None
Groin pain	3 patients complained of groin pain. It was resolved during the 6 months follow-up period	None
Trendelenburg gait	None	3 patients continued to have a Trendelenburg gait that was resolved within 6 months after surgery
Infection	None	One patient had deep infection during the early postoperative period that required operative debridement/irrigation and liner exchange along with postoperative intravenous antibiotics. No recurrence of infection at two years of follow-up
Deep venous thrombosis	None	None
Intraoperative or Postoperative blood transfusion	None	None
Numbness in the lateral femoral cutaneous nerve	8 patients	None
Delayed superficial wound healing	Five patients had delayed wound healing in the proximal part of the anterior approach incision. All patients had healed within 4 weeks post-surgery with local wound care	One patient had delayed wound healing. The patient healed after 3 weeks post-surgery

One patient in the DL group had early deep infection and required incision, drainage and polyethylene exchange. This patient had no recurrence of infection up to 2 years of follow-up.

## Discussion

In recent years, some reports highlighted higher THA complication rates when performed via DA hip approach when compared to posterolateral or DL hip approach [13–17]. These complications included higher infection risk, femoral nerve injury, fractures and early femoral stem loosening. However, these studies did not report prior surgeon’s training, learning curve and volume. In this study, all patients had their THA performed by the same surgeon during the 1^st^ year of practice and after a one year of fellowship training in a high-volume center to perform THA via both DA and DL hip approaches. The only major complication occurred in the DL group in which one patient required incision, drainage and polyethylene exchange due to early deep infection. A recent study found younger surgeons (less than 45 years) had higher overall complications when compared to middle-aged surgeons (45–55) after THA. However, when low volume surgeons (perform less than 35 cases per year) were excluded, the authors’ found volume was more important than the surgeon’s age [18]. The impact of fellowship training on total joint arthroplasty has been studied and shown to provide better surgical and functional outcomes when compared to non-fellowship trained surgeons [19]. These reports along with our findings likely reflect the importance of fellowship training particularly when adapting a relatively new surgical approach or technique.

In this patient population, although there was no statistically significant difference between the two groups regarding HOOS scores at 6 week and 6 months postoperatively, there was a trend toward better scores in the DA group. Similarly, there was a trend toward shorter hospital stay (less than 24 h), fewer opioids refills postoperatively, fewer patients requiring walking aids at one week postoperatively and lower number of LLD outliers in the DA group. While none of these findings were statistically significant, there is support in the literature that these differences are real. Wang et al. [20] conducted a Metanalysis of 5 randomized controlled trials (RCTs) comparing DA hip approach with DL hip approach. The authors found patients who underwent DA had better pain relief postoperatively and better early functional outcomes. They, however, found no difference between the two approaches with regard LOS and long term functional outcomes.

Patients in the DA group had shorter operative time (83 ± 17 min) when compared to the DL group (93 ± 24 min) with *p* = 0.03. This could be explained by the longer closure time that is required for the DL group. There are multiple soft tissue layers (hip capsule, gluteus medius tendon repair, vastus lateralis fascia, tensor fascia repair, subcutaneous tissues and skin) that require closures during the DL hip approach when compared to the DA hip approach (fascial layer, subcutaneous tissues and skin only). However, since DA patients had their surgeries performed during the second half of 1^st^ year of practice, it is also possible that the operating team and the surgeon became more efficient with the surgical steps and flow when compared to the 1st half of the 1^st^ year. The literature showed contradictory results regarding operative time. In a recent randomized prospective study [21], the authors found no difference in operative time between DA and DL hip approached in THA. While others [16, 22] showed longer operative time with the DA approach. Again, these findings may indirectly reflect the variable levels of learning curve and surgeon’s experience. Shorter operative time is linked to less postoperative infection risk. Wang et al. [23] found that each 20 min increase in operative time during joint replacement procedures was associated with 25% increase in postoperative infection. This is an important and often underappreciated risk factor for infection.

There have been unique minor complications related to the DA group. Proximal wound delayed healing was encountered in five patients compared to one patient in the DL group. While this can happen with any surgical approach, the higher frequency of this complication been reported in the literature in DA hip approach. Jahng et al. [24] reported that 11.5% of 651 DA hips experienced wound complications. The authors and others [25] found patients host factors such as diabetes and obesity as independent risk factors. However, we believe that are some technical factors could also contribute to such complication. The retraction force on the proximal incision and/or skin damage during the femoral broaching of the femur is a risk factor that is not reported in the literature. A placement of wet sponge underneath the retractors and meticulous broaching technique have minimized the occurrence of this problem in subsequent cases.

It was noticed that 2 femora had varus stem alignment in the DA group and one stem was in varus in the DL group. None of these patients has had femoral stem loosening or subsidence during the 1^st^ two years. While placing the femoral stems in varus is generally avoided, varus alignment has not been shown in the literature to lead to adverse outcomes when standard cementless proximally coated femoral stems were used [26, 27]. However, varus stem alignment can be a sign of suboptimal exposure. By focusing on better exposure, this issue disappeared in our patients. An alternative choice is to employ intraoperative fluoroscopy as a way to minimize alignment and sizing issues. This was done in 9 patients (5 in DA group and 4 in DL group during the 1^st^ 10 cases of each group) at the time of femoral trialing with the broach in place. It lead to a broach change in only one case in this series with no clear effect on the clinical outcome of the surgical procedure. We feel that the most important way to ensure proper alignment and sizing on the femoral side is to focus on a better exposure. Routine fluoroscopy, while an option, was not helpful in this series**.** This finding was echoed previously by Tischlar et al. [28]. The authors found routine fluoroscopy (N = 178) did not improve components positing or decreased complications in THA when compared to the freehand technique (n = 163) in high volume center.

This study has some limitations. It is a retrospective study. Nevertheless, the selection bias was minimized since all patients underwent THA via DL approach during the 1^st^ 5 month of practice and then followed by DA approach for the rest of the 1^st^ year. Due to the relatively small sample size in each group, it is possible that the study is underpowered to show statistically significant results when comparing certain variables due to low event rates in both groups. However, it would be difficult to achieve a larger sample size and volume during the 1^st^ year of practice.

In conclusion, both DA and DL approaches have resulted in satisfactory results and acceptable complication rates at short term follow-up when performed by a fellowship trained surgeon using the same perioperative protocols during the 1st year of practice. The selection of surgical approach remains at the surgeon’s discretion with potential benefits of the DA hip approach over the DL hip approach during the early perioperative period.
